# Drones as internet of video things front-end sensors: challenges and opportunities

**DOI:** 10.1007/s43926-021-00014-7

**Published:** 2021-05-10

**Authors:** Chang Wen Chen

**Affiliations:** University At Buffalo, State University of New York, Buffalo, 14260 USA

## Abstract

Internet of Video Things (IoVT) has become an emerging class of IoT systems that are equipped with visual sensors at the front end. Most of such visual sensors are fixed one whereas the drones are considered flying IoT nodes capable of capturing visual data continuously while flying over the targets of interest. With such a dynamic operational mode, we can imagine significant technical challenges in sensor data acquisition, information transmission, and knowledge extraction. This paper will begin with an analysis on some unique characteristics of IoVT systems with drones as its front end sensors. We shall then discuss several inherent technical challenges for designing drone-based IoVT systems. Furthermore, we will present major opportunities to adopt drone-based IoVT in several contemporary applications. Finally, we conclude this paper with a summary and an outlook for future research directions.

## Introduction

The development of Internet of Things (IoT) has been progressing rapidly in recent years, evolving from conventional IoT applications in which only sparse and scalar sensor data are acquired, to the emerging Internet of Video Things (IoVT) [1] applications consisting of massive number of visual sensors. With visual sensors, IoVT systems exhibit some unique characteristics in terms of information sensing, transmission, storage, analysis, and integration. Among IoVT systems, the most well-known one is the surveillance system that acquire the sensor data via conventional frame cameras internetworked to provide automatic detection and recognition by the system. However, IoVT systems employing drones as their front end sensors have seen their explosive deployment recently in several innovative applications, including supply-chain delivery, search and rescue, and athletes’ movement tracking.

**Fig. 1:**
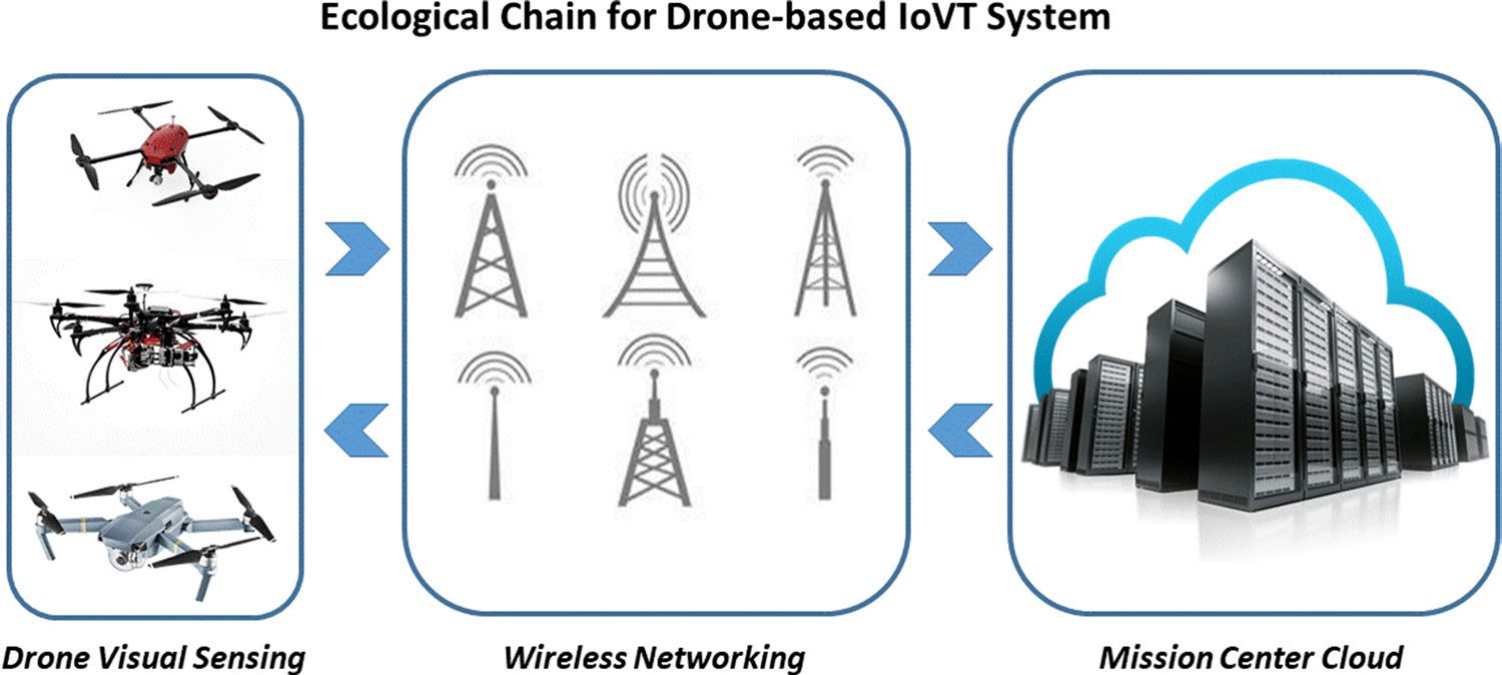
Three functional blocks of a drone-based IoVT system

What makes IoVT fundamentally different from conventional IoT is its front end sensor capable of acquiring image and/or video data providing 2D or 3D rendition of the physical world. What makes IoVT systems equipped with drone as their front-end sensor fundamentally different from other IoVT systems is their capability to proactively fly over targets of interest and acquire dynamic imagery data [2]. In general, a drone-based IoVT system consists of three major distinct functional blocks with the ecological chair of such dynamic system, namely, drone visual sensing, pervasive wireless networking, and cloud-based analytics at the mission center. These three functional blocks are illustrated in Fig. 1. Notice that these functional blocks are not one-way chains that moving visual data from front-end sensors to mission center clouds. This system facilitates the command and control from mission center to be pushed down to frontend flying drones for controlling of the flight ae well as the data acquisition. The feedback from the networking functional block also facilitate the implementation of edge computing strategy for coordinating the embedded processing and wireless networking. The unique ability to deploy the drones preemptively to the designated area and to collect the visual data dynamically indeed creates new opportunities for IoVT applications. However, such unique IoVT systems also pose new technical challenges in terms of stable visual sensing, robust communication and networking, and integrated decision making and actuation.

In this article, we shall first analyze several unique characteristics of the emerging drone-based IoVT systems. It is these unique characteristics that facilitate the new capabilities of drone-based IoVT systems when the front-end visual sensors are flying drones. However, this new class of IoVT systems also pose significant technical challenges throughout the entire IoVT system chain from frontend sensing to information networking, and to backend analysis and decision making. We shall present in detail some inherent limitations triggering these technical challenges as well as potential approaches to overcome them in order to develop a robust and efficient drone-based IoVT system. We will also illustrate a few major opportunities to deploy drone-based IoVT systems in contemporary applications to achieve new capabilities that cannot be accomplished using other types of front-end sensors.

The primary motivation for this article is to introduce the emerging scenarios in which drones are adopted as front-end visual sensors for acquiring image and video data that cannot be accomplished by other type of visual sensors. This article is not intended to provide comprehensive survey of drone-based applications in a wide range of potential usage cases. Rather, we limit the application scenarios in which drones are adopted to serve as the front-end sensor within the emerging systems under the IoVT architecture [1].

The contributions of this article is three-fold: First, this article presents an analysis of unique characteristics in terms of the inherent constraints when the drones are adopted as IoVT frontend visual sensors. These constraints include the nature of the undertaking as spontaneous missions, the need for lower power frontend unit onboard flying drone, the requirement of stringent networking capability of the link between drones and the control center, and limited onboard processing and storage capacities.

Second, this article recognizes several technical challenges among three subsystems of the overall IoVT architecture. At the frontend data acquisition subsystem, stable and robust visual sensing needs to be carefully designed in order to maintain stable data acquisition while flying, robust sensing under varying ambient environment, and onboard processing and analytic to ensure real-time mission control. At the communication and networking subsystem, reliable and low latency transmission of volumetric visual sensor data is required, including both pre-planned missions when the networking infrastructure is known ahead of time and emergency missions while the networking capacity is unknown and needs to be discovered while the drone is flying to the designated locations. At the cloud-based central control subsystem, there is often a need to use drones as edge computing nodes to help the cloud center to implement 3C strategy for optimal integration of sensor data for overall mission control under the emerging edge-fog-cloud architecture.

Third, this article also identifies two application opportunities when drone-based IoVT system can take full advantage of its niche visual sensing capabilities and its flexible deployment potentials. These emerging applications include fast package delivery for supply chain and e-commence as well as dynamic tracking and filming of athletic in fast motion and dancers in spinning action. Only drone-based IoVT systems can accomplish such demanding application scenarios.

## Characteristics of drone as IoVT frontend sensors

For an IoVT system equipped with drones as its frontend sensors, there are several unique characteristics that need to be carefully examined in the design of such IoVT system. Unlike conventional IoT or even IoVT systems, drones usually are deployed via flying means to the target areas to acquire visual renditions of the target and possible their movement through tracking. Such unique deployment and data acquisition instrument demand special configuration of IoT design. With this configuration, the characteristics of drone-based IoVT range from spontaneous mission scenarios to harsh power constraints, and from on-board processing requirements to stringent networking requirements, and from robust flight control to seamlessly integrated 3C strategies. We shall explain in details these characteristics in the following.

### Spontaneous mission scenarios

For conventional IoT applications, the locations under surveillance is often fixed and preselected before sensor deployment. The IoT sensors are often embedded into the environment under surveillance and remain at the deployed position throughout the data acquisition process. For drone-based IoVT applications, the location for drone deployment is often unknown until the mission is determined. The drones often need to fly from their storage camp to mission area with an effective flight path planning. This is true for special cases in search and rescue mission under disaster management scenario [3]. This is also true for drone delivery of packages in contemporary supply-chain applications. There are two fundamental differences in the deployment of frontend sensor between conventional IoT applications and emerging applications that demand spontaneous scenarios. First, the drone as IoT sensor is capable of collecting dynamic visual information over relatively large terrain unknown to the drones before the mission is determined. Second, the flying drones are expected to work even when the potential target under surveillance is in motion which requires the drones to detect, track and follow the moving target. The spontaneous nature of the flying drone based IoVT poses significant challenges in how the sensor data are collected, how the sensor data are networked, and how effective actuations may be designed and applied based on the results of sensor data analysis.

### Low power frontend units

The IoVT systems based on drones need to be powered by battery to facilitate their flexible flying capability. However, multiple power hungry operations are inherent to the drone-based IoVT systems for them to work properly. For battery-supplied flying drones, these battery hungry operations are embedded throughout the entire system chain from frontend acquisition of visual sensor data, to the networking layers for transporting sensor data as well as onboard processing results, and to wireless reception of feedback of information analytic results and possible actuation command from backend system [2]. Unlike the conventional IoT applications, visual sensing onboard drones consumes substantially more energy due to volumetric nature of visual data and wireless connectivity needed for flying nodes. To facilitate fast response required under spontaneous mission scenario, onboard processing of acquired visual data is often necessary. This imposes additional power constraint on the battery capacity of the flying drone. Furthermore, full networking connectivity of high bandwidth for transmitting just-in-time visual data and onboard processing results is another major source of power consumption. Finally, there will be needs for receiving wirelessly the feedback on information analytic results and actuation commands from backend systems.

### Stringent networking requirements

Drone-based IoVT systems may be deployed with full networking support for well-planned missions supported by significant communication infrastructure. However, drone-based IoVT systems are often deployed for those spontaneous missions without networking infrastructure support. It is particularly true that the networking infrastructure is severely, or even completely, damaged under the scenarios of search and rescue missions required for disaster management [4]. In either cases, there is need for uploading high volume visual sensor data and possibly onboard processing results to the control center for necessary real-time interventions. This will require high bandwidth transmission between drones and their control center. For the nature of drone deployment, wireless networking is often the only means for properly transmitting and receiving data to and from the control center. Depending on the distance between drone deployment site and control center, intermediate relay stations may be needed which shall introduce additional latency and more transmission errors. One possible handy relay stations may be smartphones carried by a human agent within the distance of transmission [5] which will transmit the captured visual sensor data to the control center and may also relay the command and control to the flying drones for possible actuations. In any case, the volumetric nature of the visual data demands stringent networking capability in both bandwidth and latency requirements.

### Limited on-board processing/storage

For real-time missions that require the visual data to be instantly uploaded to the mission center for possible decision and actuation, on-board processing is often mandatory with embedded visual analytic algorithms running while acquiring visual sensor data. However, battery operated drone systems need to guarantee the flight time of the mission by trading off the energy consumption between on-board processing and flying maneuver. Only limited on-board processing capacity is allowed for the analytic results to be transmitted to the mission center. Therefore, most visual sensor data required during the mission may need to be saved in the on-board storage device for retrieval after the mission is completed. The need for on-board storage is particularly true for cases when real-time on-board processing is not necessary while the analytics of the visual sensor data can be off-loaded to cloud center for high performance processing [6, 7]. Current practice for on-board data storage has two possible solutions. A variety of drones adopted as an IoVT front-end sensor nodes may possess very limited original storage capability. To accommodate IoVT applications, additional memory expansion options like Secure Digital or micro-SD will be selected with easy insertion and removal so as to increase their original memory capacity to store visual sensor data. Nevertheless, for some long duration drone flight, such additional memory may still be inadequate for storing all visual sensor data. There is need to strike the balance between on-board processing/coding capability to reduce the data volume and storage capacity to carry the visual sensor data back to cloud center for full scale processing and analytics.

## Technical challenges in adopting drones as IoVT frontend sensor nodes

The characteristics of drone as IoVT frontend sensors as outlined above pose several technical challenges at various stage of drone-based mission, ranging from flight planning for visual data acquisition, to seamless communication and networking, and finally to information integration at the mission center. In the following, we shall present these challenges in more detail to demonstrate the level of technical complexity when designing high performance drone-based IoVT applications.

### Stable and robust visual sensing for high quality sensor data acquisition

To ensure that a drone-based IoVT application can maintain high quality sensor data acquisition even under some extreme conditions or under varying ambient environments, we are facing technical challenges in: (1) maintaining stability of drone while flying, (2) upholding robustness while acquiring visual sensor data, and (3) on-board analytic for real-time applications. We will need to first address the technical issues related to the flying dynamics of the drones that are in transit from control center to the mission locations. We will also need to address the technical issues related to robust acquisition of visual data under varying ambient environment which may significantly impact the quality of data acquisition. Finally, depending on the nature of the drone mission, on-board data analytics may need to be implemented to ensure just-in-time actuations based on the analytic results.

**Fig. 2:**
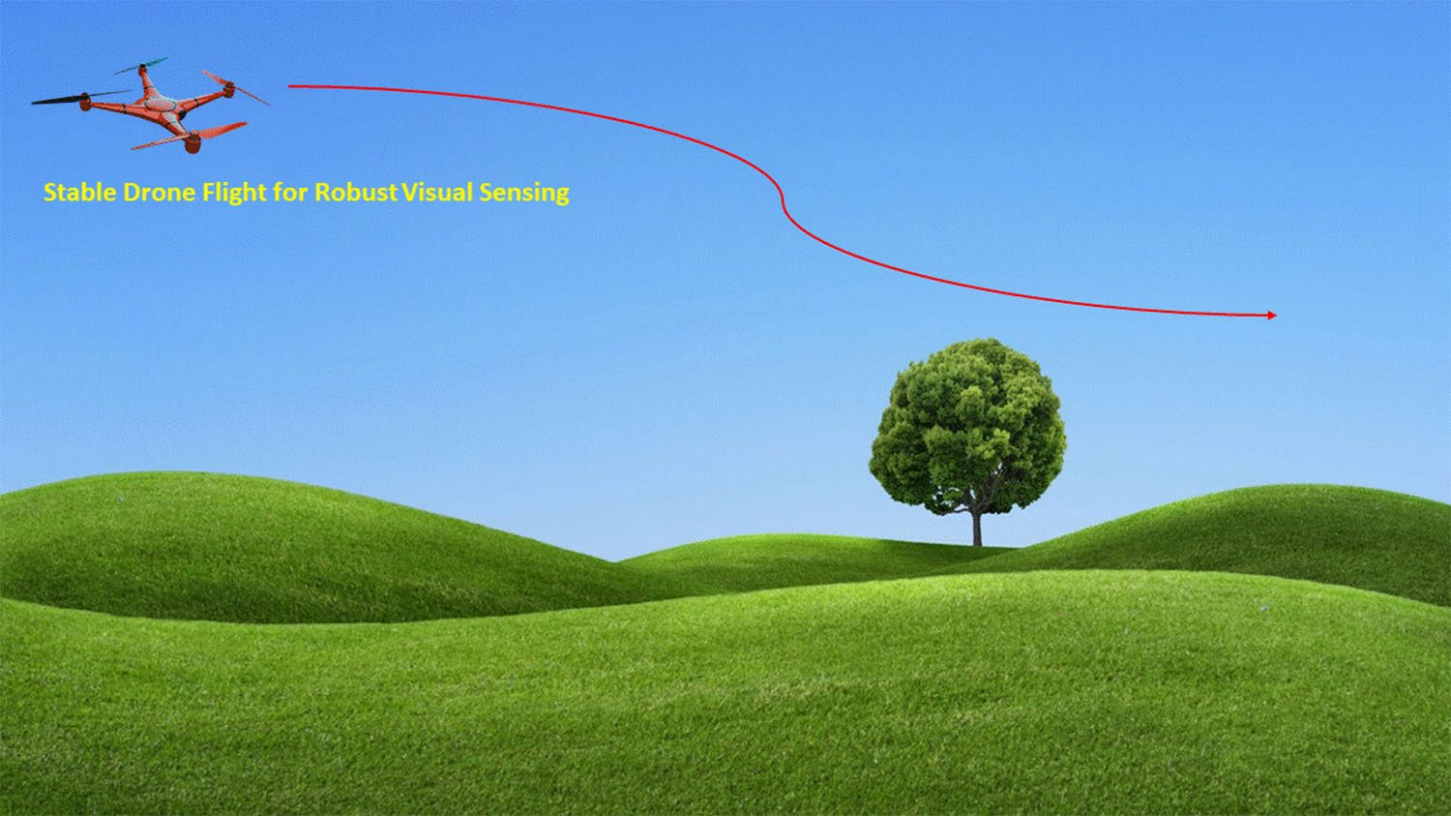
An illustration of stable drone flight for robust visual sensing

For any mission that uses drone to acquire visual data at a location that mandate the flying of the drone from its control center to the location of interests, the flying during its mission must have access to communications and navigation signals for the duration of the mission in order to keep the flight path as defined by the pre-mission planning. Such an drone flight control scenario is illustrated in Fig. 2. Flight path planning at the pre-mission stage typically includes consideration of where and when these signals are available and where and when they may be blocked. However, sometimes communication or navigation signals are blocked unexpectedly because of jamming or because of unknown terrain changes. Maintaining the stability of the flying drone under unexpected scenario is very important in order to accurately send the drone to the desired location of interest. Autonomous or semi-autonomous flight control is desired especially when the location of interest is sufficiently far away from mission centers [8].

The design of autonomous or semi-autonomous flight control is heavily dependent on both communication and navigation signals and various on-board sensing data that can be processed in real-time to calibrate the instant position of the drone and to control the flight of the drone [9]. In additional to navigational signal such as GPS, other type of sensor data on-board the flying drone including video cameras, gyroscope, and infrared sensors shall need to be integrated to provide more accurate flight positions relative to the terrains of flying-over zones. The sensor fusion approach has been studied for various applications. For example, in the case of forest-use drone, long and stable flight is necessary for highly efficient and safe forest survey [8]. In addition to making use of various sensing data, modeling of pitch propeller for proper control of drone flight is also needed. Furthermore, advanced control for attitude-hold flight is also necessary for missions that require stationary acquisition of visual sensor data in order to detect and track target of interest. When the drones are deployed in a dynamically changing environment, pre-mission flight path planning would in adequate because possible obstacles may present in the flight path after the mission planning and the drone may depart from the nominal mission path. Under these scenarios, the flying drones are required to be able to detect and avoid unexpected obstacles to ensure safe and stable flight. Multi-sensor fusion is often desired to implement such a highly sophisticated mission [10].

Similar principles of flight control can also be applied during the data acquisition process which may require the drones to follow certain pre-planned sensing path. The sensing path may also include a stationary holding of the drone while executing the data acquisition. Robust sensor data acquisition is highly correlated with the positioning of the drone during the flight. However, the pre-planned sensing path may need to be altered during the mission for the events when the drone is used for tracking moving targets, or for disaster management of search and rescue. Usually, a fusion of sensor data from multiple sources is needed to ensure the accuracy of the event detection [end-to-end]. The actuation of drone flight path adjustment will start and the adjustment will continue until the desired position and attitude is achieved. For all these cases, on-board sensor data analytic algorithms will need to be activated to achieve desired intervention and actuation.

### Robust communication and networking during entire flying mission

One major characteristic of visual sensor data acquired by drones is its volumetric nature which shall impose stringent requirement for communication and networking during the entire drone flying mission. This is particularly true for real-time application missions when the visual sensor data need to be transported instantly to the mission center for cloud-based processing and analysis. The combination of high bandwidth and low latency throughout the mission period demands robust communication and networking for drone-based IoVT applications.

**Fig. 3:**
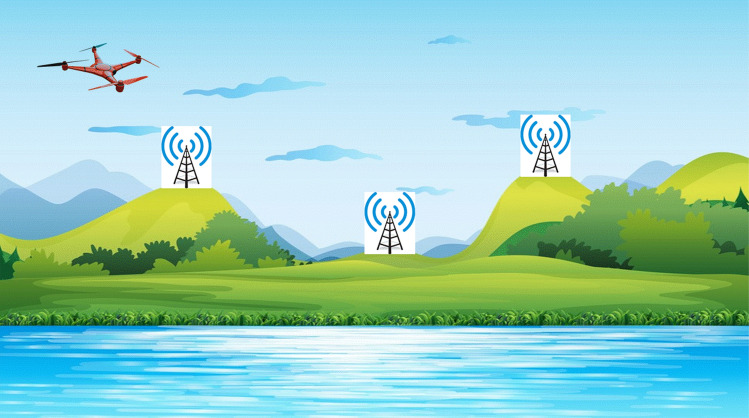
An illustration of drone mission communication and networking during flying

Among various scenarios, many missions can be scheduled to follow pre-computed flight path in which communication and networking capability of the drones along the path becomes one major parameter for quality of service. We call these regular missions in which known wireless networking infrastructures are present along the path for drone networking to maintain desired real-time communication between flying drone and mission center during both flight control and visual data acquisition. Such a drone mission communication and networking scenario is illustrated in Fig. 3. Typical regular missions include surveying tasks in which the terrain characteristics of area under surveying and locations of the networking infrastructures are both known during mission planning. It is possible to calculate the communication and networking capability and predict the quality-of-service level based on both flight control parameters and known networking infrastructures including location of the relay stations and communication channel capacities of them. As the drones in flying mode are connecting to intermediate stations wirelessly, the channel capacity of these stations is dynamically varying over time and space. Therefore, adaptive algorithms for transmitting flight control signal as well as acquired visual sensor data need to be designed to combat significant bandwidth fluctuation of the drone networking. Such adaptive algorithms should be developed to guarantee robust transmission of volumetric visual sensor data when the adversary effects resulting from channel impairment of the wireless communication can be minimized and minimum bandwidth for data transmission can be maintained throughout the mission process.

In the case when the drone mission is called for emergency management, the communication and networking infrastructures are either unknown at the time of mission planning or even substantially damaged by natural disaster. The technical challenges to maintain robust communication between flying drones and mission center become much greater. Under such scenarios, there is a need for drone to actively discover the terrain structure as well as the networking possibility along the way while flying to the area of interest. In particular, drone swarm consisting of multiple drones can be deployed to form temporary wireless network [11] for critical command and control as well as for just-in-time visual sensor data transmission. Some of these drones can be deployed for visual sensor data acquisition while other drones may be deployed for the purpose of serving as hotspots for networking between the drone swarm and the mission center. There have been some recent efforts in designing and deploying drone-borne wireless networks for disaster management that employing various networking technologies [12], including flying mesh networks, wireless multi-hop networks, mobile ad hoc networks, and software defined radio [13]. The performance of these drone-based wireless networking designed are subject to limited energy supply by battery [14]. Accurate visual mapping of the disaster area terrain is vitally important for organizing search and rescue and the transmission of such data to mission center needs to be guaranteed. For drones that are employed as an IoVT frontend node, the volumetric nature of the camera data acquired for visual mapping demands high performance and low latency networking capability to achieve an effective disaster management, especially for search and rescue missions.

The operational mode of drone-based IoVT systems will have major impact in the communication and networking capability of the such systems. Drone-based IoVT applications often involve two major operational modes: (1) visual data acquisition and on-board storage, and (2) on-board processing and analytics for quick action such as tracking. In the case of first operational mode, the sensor data may be uploaded to cloud center for processing via wireless networking or upon the completion of the fly mission. In the case of second operational mode, it is required for the drone to have on-board processing and analytics capability for quick action such as tracking while still uploading the visual sensor data to cloud center via wireless networking. As the drones are battery operated during mission, there is need for the overall system design to consider a trade-off between the energy consumed for on-board processing and the energy consumed for wireless networking. It is absolutely necessary to ensure that the drones have enough energy supply until they complete the flying mission and return to base station. It is this second operational mode that creates the opportunity for drone-based IoVT applications to jointly consider the impact of energy consumption from both computing and networking aspects.

### Drone as an edge computing node for integrated 3C strategy

**Fig. 4:**
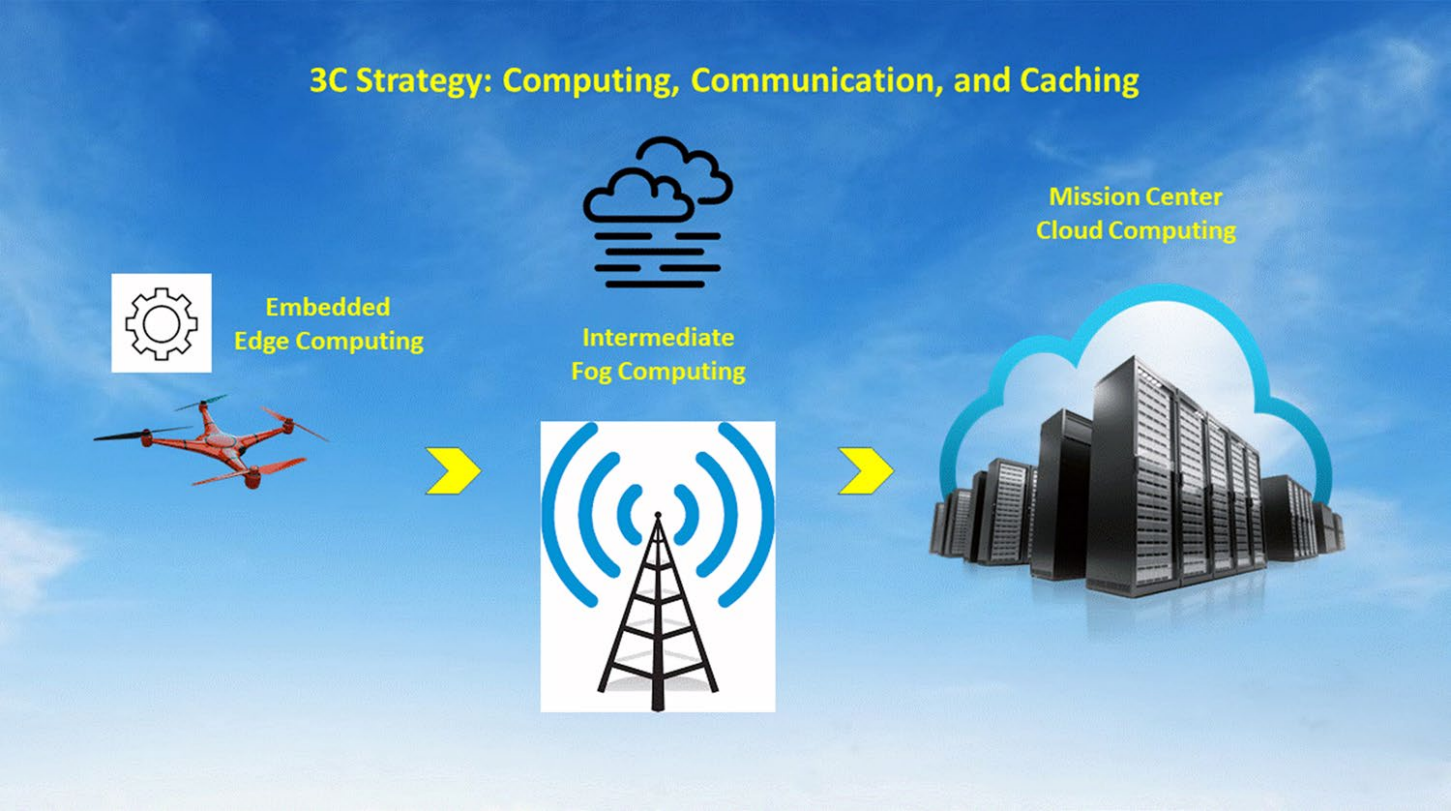
An illustration of 3C (Computing, Communication, and Caching) strategy

The ultimate objective for drone-based IoVT system is to accomplish its mission under various resource constraints throughout its ecological chain of operations from frontend drone sensing, to networking linking drone and mission center, and to cloud-based computing at the mission center. Within this ecological chain, there are three basic pillar technologies involved at various stages: computing, communication, and caching, each of them shall compete for limited energy resource on-board the drones as they are all powered by battery. The convergence of computing, communication, and caching has seen its accelerated development in recent years, especially in the area of Internet of Things [15]. This convergence is called 3C strategy and its principle has been successfully adopted for numerous information and communication systems. An illustration of such strategy is shown in Fig. 4. For resource constrained applications such as drone-based IoVT, it is critical for to adopt a proper 3C strategy designed for a particular application scenario in order to maximize the overall IoVT system performance. More importantly, in the case of drone-based applications, the power consumption for 3C strategy are severely constrained by the power consumption on-board the drones needed for maintaining the drone flight throughout the entire mission.

For drone-based IoVT applications, the energy constrained and dynamic flying nature of the drones pose additional technological challenges. Some of the inherent challenges may be alleviated by deploying additional drones to form drone swarm to complete the mission collaboratively. For single drone mission, the 3C operations can be allocated between drone and the cloud at its mission center. As indicated in a recent position paper about IoVT [1], it is important to select an intermediate networking layers for implementing the 3C strategy in order to optimize for overall system performance. For drone-based IoVT applications, the ultimate data sinks will be the cloud at the mission center which is usually located with physically away from drone nodes. This setting requires wireless networking to transport the visual sensor data for extensive computation on them. From the network architecture point of view, there may exist several layers between the frontend nodes and the cloud centers and each layer has its own unique capability to support 3C. A recent development in edge-fog-cloud architecture is ideal to facilitate an opportunity for exploiting the 3C strategy. This is particularly true for the scenario when a fleet of drones are working collectively and some of the drones shall be designated as edge computing nodes to help collaborating drone nodes offloading their computation tasks for low latency and highly accurate intermediate results for on-board decision making. Such 3C strategy has been successfully applied to visual navigation and mapping missions in which edge computing has been adopted to offload computational task and has shown its effectiveness [16].

Among recent efforts, the fog-aided approach [17] has also been demonstrated to achieve desired performance by employ fog nodes to provide computing resources for the delay-sensitive tasks offloaded from drones. This approach considers both the task allocation that distributes tasks to different fog nodes and the flying control which adjusts the drone’s flying speed to minimize the completion time of flying drone constrained by its battery capacity [17]. Broadly speaking, fog computing is a collection of operations, including computing, networking, storage, control, and acceleration and may be located anywhere within the cloud to things continuum. The major difference between edge computing and fog computing is that the edge computing is usually limited to mainly computing operations at or near the frontend device. The design of edge computing can be fully integrated with the design of frontend sensing system, including both single drone mission and drone swarm missions. In the case of visual navigation and mapping that multiple collaborative drones are deployed, the major design principle is based on the trade-off between different operations which may be carried out computation locally on-board sensing drone, or offload to a collaborative drone as edge computing server, or upload to the remote cloud at the mission center [16].

Drone-based edge computing has also been applied to disaster emergency management to construct a temporary mobile edge infrastructure to facilitate critically needed communication and networking capability [18, 19]. After major disaster, normal communication and networking infrastructures are usually seriously damaged or even completely collapsed. Drones can be relatively easily deployed to create mobile edge infrastructures within a short period of time to help search and rescue operations as well as adversely affected population to maintain their communication capabilities [18]. It has been shown in [19] that a mobile edge infrastructure can be constructed quickly using buses-and-drones mixture to aid the critical disaster management mission. The buses in this system not only serves as mobile edge computing nodes, but also provides steady power resources for charging the flying drones. The combination of buses and drones enables the mobility of edge nodes as well as the power supply of the flying drones to form a stable temporary communication and networking infrastructure for disaster management. Drone-based edge nodes are employed to extend the coverage of the temporary communication network to include the hard-to-reach areas caused by disaster. We can conclude from these prior studies that drone-based edge computing principle can be applied beyond the primary usage in drone-based IoVT frontend sensor nodes discussed in this paper and become a key component for constructing mobile edge infrastructure for disaster management.

## Emerging opportunities for drones as IoVT frontend sensor nodes

Rapid developments in various areas of information and communication technologies, as well as in artificial intelligence and machine learning technologies, have opened up many emerging opportunities for adopting drones as IoVT frontend sensor nodes. One distinct advantage of drones with visual sensing capability is that visual sensing can provide unique and holistic insights that conventional IoT sensors are unable to obtain. This advantage shall empower drone-based IoVT system to penetrate into new application domains that require quick identification of visual patterns for decision and actuation. Another distinct advantage of drones is its flying capability that enables the sensing node to become mobile and deployable to areas of interest quickly through autonomous flight control. The combination of these two distinct advantages in visual sensing and fast mobile deployment make the drones uniquely applicable to various challenging scenarios. We have described some application scenarios such as search and rescue in disaster management when discussing the technical challenges for adopting drones as IoVT frontend sensing nodes. Here we present two interesting application opportunities that take full advantage of drone’s unique characteristics.

### Fast package delivery in supply-chain and e-commerce applications

Fast and reliable package delivery is crucial for supply-chain and e-commerce applications. This is particularly true for last-mile logistics in which the recipients usually need to be verified upon package arrival. Drone-based IoVT system is indeed capable of verifying the recipients via frontend visual sensing and embedded analytics on board the drones. More importantly, with their flying nature, the flight path for delivery is not constrained by street traffic on the ground which often significantly slow down delivery trucks in conventional means of package delivery. The flight path can be pre-computed ahead of time at the local distribution center to substantially speed up the last-mile delivery.

Currently, major corporations such as Amazon, Walmart, UPS, Google and other global companies are investing heavily in developing drone delivery. Their pilot programs based on such disruptive technology have shown promising initial success [20]. Drone-based package delivery enables both reliable recipient verification and fast delivery, two crucial advantages in deploying this disruptive technology. Moreover, these autonomously flying drones can save substantial labor and ground transportation costs that have been major portion of expenses in e-commerce companies [21]. However, one significant challenge in drone-based package delivery is how to overcome the potential jamming of the flying drones in urban low level airspace as the flying drone density for commercial package delivery shall become very high when more and more commercial companies adopt the drone-based package delivery. This issue was studied recently in five European counties and several major metropolitan areas and their results suggest that hourly traffic density may easily exceed today’s aircraft traffic of 10,000 per day by six-fold for a typical major metropolitan area such as Paris [estimation]. To be able to accommodate the potential crowding traffic within the low level airspace of a metropolitan, a robust airspace management system needs to be developed in order to meet the requirements of the commercial package delivery. One positive impact of adopting drone-based package delivery is its great potential in substantially reducing the ground traffic congestion as many current delivery trucks are replaced by flying drones. This in turn will have positive impact in air pollution reduction because of the number of parcel delivery trucks are also reduced accordingly.

One particularly important applications of drones for package delivery is in the healthcare sector when medical products need to be dispatched to people in need in a timely fashion [21]. Unlike the regular package delivery, the delivery of medical products is usually more sensitive to the arrival time with stringent delay constraints. This is particularly true for delivering test samples from the patients, such as blood samples, for timely processing at a location away from the clinic. The flying capability of the drones enables fast and accurate delivery with advanced path planning. Based on the study carried out for the city of Rotterdam, it has been found that drone-based medical product delivery is able to improve several key performance indicators in payload utilization, service time, environmental benefits and cost reduction [21]. Moreover, the drones have also been utilized for true medical emergency scenarios, such as cardiac arrest, to deliver the necessary lifesaving packages. In the case of cardiac arrest, it is extremely important to have an automated external defibrillator (AED) at the location of an out-of-hospital cardiac arrest as soon as possible to save the life of patient [22]. Comparing with conventional emergency medical services provided by ground vehicles, it has been shown that the drone-based approach can achieve 80.1% of the cardiac arrest demand being reached within one minute while the conventional practice can only achieve 4.3% of the cardiac arrest within one minute from the emergency call. This significantly reduce the time from demand to the arrival of AED [22, 23].

This year marks the most turmoil around the world due to the COVID-19 pandemic. When people are requested to stay under quarantine, there is increasing needs for the delivery of daily needs to individuals who are home bound. We have witnessed some innovative use of drones for delivering medical and healthcare supplies, as well as foods and other supplies, to serve those who are under stressful medical quarantine [24]. One additional advantage for drone-based delivery of packages of essential needs is its potential in eliminating contaminations through the minimization of human contact during the entire process. With its flying capability, it is also possible for drones to reach some less accessible regions to enhance the quality of life for the disadvantaged population [24].

### Dynamic tracking and filming of athletes in motion or dancers in action

Another interesting application opportunity for drones as IoVT frontend sensing node is for the tracking and video coverage of athletes in motion or dancers in action. Different from product package delivering for regular e-commerce or for life-saving healthcare applications, the tracking and filming of athletes and dancers have been developed to overcome some inherent challenges in these events that it may be impossible to cameraman to follow on foot due to fast motion or due to inaccessible venues [25]. The drones with embedded sensor data analytic algorithms shall be able to track the subjects and fly the drones along the tracking direction and speed even the subjects under tracking are in high motion. The control of drone flight based on the tracking results maintains the stability of flying and can provide excellent video quality for viewing by the remote audience.

There are two main objectives for drone application in sports and entertainment. The first one is its sport and artistic communication objective for a more realistic and more close-up filming of the athletes and dancers for the remote viewers [26]. Through tracking the athletes or dancer dynamically within a short distance, the videos from drones can bring some immersive feelings as the cameras from the drone appear to be rolling along with the subjects in motion. Such immersive feelings can only be achieved with close-up video while the drones are also able to move along with the subject’s high motion. Furthermore, the drone-based IoVT cameras can also be commissioned to fly above the outdoor sporting site to observe from an overview distance to grasp the spatial, tactical and situational dimensions of the events on sporting landscapes [26]. The second one is its training objective to understand the dynamics of a top athlete in high motion or the overall flow of choreograph by a top dancer in action. Under these scenarios, There have been several prior research efforts in tracking player motion for the purpose of sport training as well as for the purpose of understanding game strategy in sport, especially in team sporting events [4, 5]. There efforts have been shown to offer significant inside by processing and analyzing videos recorded by drones with well-designed flight mission and configuration. For various application scenarios, there may be a need to deploy more than one drone to record the action from multiple viewing angles for more immersive reconstructions for sport communication or an improved understanding of athletes or dancers in motion.

In the case of understanding sports strategy formulation, the goal is to provide a competitive advantage to a team or individual by analyzing a collection of statistics that can be derived from applying video processing and analysis techniques to the drone recording. In particular, an automated player detection and tracking algorithm can be designed so that the drone-recorded videos enable better understanding of play formulation and sport tactics. For the study case reported in [4], the researchers have solve one particular technical challenge in avoiding occlusion of players after the drone was designed to be on flight at overhead positons as much as possible.

In the case of providing better training of athletes via drone recording of video in an autonomous fashion, the flying path of drones is controlled by the features extracted from the video [5]. There are various features that may be adopted for tracking the athletes via video analytics. However, the need to control the drone flight instantly would impose the requirement that the computation of vision features can be implemented on-board the flying drone in real-time. For the study case reported in [5], as this is for one player scenario, color was adopted as a stable and easy to detect feature for tracking the player’s motion during the training session. This way, the system can track the player in real-time while the drones can be positioned to shot the video from best possible angle for subsequent analysis and understanding.

Another interesting sport related application scenario is the use of drones as an effective transportation means to provide first-aid materials to the scene for outdoor sport activities [27, 28]. This is particularly true for field-based sport such as golf that the players may be substantially away from the managing center where the first-aid materials are usually located. The drone-based first-aid delivery can achieve both speed and accurate location even when the ground terrain may not be smooth for vehicle transportation. Timely delivery of first-aid materials, including AED packages, to the accident location will make a critical difference when life threatening event such as heart attack occurs to any of the sport player in the outdoor field.

## Summary and looking ahead

We have presented in this paper an overview of a new class of IoVT system where the drones are deployed as the frontend visual sensors. This new class of IoVT system is fundamentally different from other IoVT system in that the frontend visual sensor nodes are in flying motion both during the flight to the areas of interest and during the visual data acquisition. This unique characteristic of drones as IoVT frontend opens up numerous unconventional applications. However, the associated technical challenges are also great and are present inherently throughout the ecological chain of this flying IoVT system. We have outlined in this paper several unique characteristics and the technical challenges within and across the three functional blocks as illustrated in Fig. 1. We have also presented two application opportunities in the future, one in package delivery under various scenarios, including e-commerce, healthcare, and even pandemic management, and another one in tracking and filming of athletes in motion for both sport communication and training purposes.

These technical challenges are actually the sources for innovation in order to construct an overall optimized IoVT system for a given application. We expect that this review will spark more innovative research to address the challenging issues arising in the future pursue of research and development in constructing drone-based IoVT system. The impact of these innovations may well go beyond the technical performance of the drone-based IoVT system. We expect that a well-designed system shall have very positive impact in the economic sense through implementing an IoVT system for smart city projects as an effective and energy efficient visual data sensing alternative to fixed camera sensing [29].

There are other important technical issues that were not covered in this article as this article focuses primarily on the application of drones as IoVT frontend sensors. Among those technical issues, security and privacy concerns are inherent in all IoVT applications because the visual sensors capture intuitive pictorial information of people and objects within the field of views of the cameras onboard drones. There are a large body of works related to security and privacy in a broad range of IoT applications. For IoVT applications, brief discussion about security and privacy was presented in [1]. Clearly, an in depth review of security and privacy for IoVT is very much desired in order to take full advantage of emerging IoVT systems with their frontend sensors equipped with visual cameras.
